# Intrapulmonary cavitating solitary fibrous tumor: A rare case report

**DOI:** 10.1016/j.radcr.2024.09.129

**Published:** 2024-10-23

**Authors:** Dyan Wahyu Kusumaningrum, Fierly Hayati

**Affiliations:** a Department of Radiology, Dr. Soetomo General Academic Hospital, Surabaya, Indonesia; b Department of Radiology, Faculty of Medicine-UNIVERSITAS AIRLANGGA, Surabaya, Indonesia

**Keywords:** Solitary fibrous tumor, SFT, Intrapulmonary, Cavitating

## Abstract

Solitary fibrous tumor (SFT) is a rare mesenchymal tumor that affects most commonly in the sixth or seventh decade of life. They account for less than 2% of all soft tissue tumors. In the thoracic region, it primarily appears in the pleura and rarely in the intrapulmonary. SFT typically presents as well-defined, oval or round, hyperdense masses. We report male 66-year-old complaint of cough, intermittent shortness of breath, and a lump in the lower right chest which has gradually increased in size. From chest MSCT results, there were cavitating lesions with solid and cystic components. FNAB and core biopsy examinations were performed. To establish a definitive diagnosis, an open biopsy followed by immunohistochemical analysis was conducted, confirming the presence of a solitary fibrous tumor (SFT).

## Introduction

Solitary fibrous tumors (SFT), previously known as “benign mesotheliomas,” are rare mesenchymal tumors that were first described by Wagner in 1870 and later characterized by Klemperer and Rabin in 1931 [1]. SFT is considered a fibroblastic tumor, it can indeed occur in various anatomical locations, it most frequently arises in the pleura. However, given its mesenchymal origin, SFT is not confined to a specific location. SFTs primarily affect adult patients, usually between 20 to 70 years of age, most commonly in the sixth or seventh decade of life. Overall, solitary fibrous tumors account for less than 2% of all soft tissue tumors [1]. In largest study, the extra meningeal SFT cases were distributed as follows: abdominal cavity 31%, limbs 29%, pleura 22%, trunk 11%, and others. In 2013 WHO classification was integrated under the SFT nomenclature the former hemangiopericytoma denomination, and in 2020 WHO classification eluded the terms of “typical” or “malignant” as typical SFT was not necessarily synonymous with benign disease [2]. Macroscopically, these tumors are well-defined, lobulated, and firm, which may grow to several centimeters in diameter and may attach via a stalk or pedicle. On cut section, solitary fibrous tumors have a fibrous, whorled appearance, with intermixed areas of cystic degeneration, calcification, hemorrhage, and necrosis [1].

## Case report

A 66-year-old male was referred from pulmonary outpatient clinic with a complaint of cough lasting for 4 months and producing clear sputum. The patient also reported intermittent shortness of breath over the past 4 months. The patient noticed a lump in the lower right chest since 4 months ago, which has gradually increased in size. He has felt intermittent chest pain over the past 2 months. The patient denied any hemoptysis or fever. He reported an 8 kg weight loss over the past 4 months. Vital signs result blood pressure 110/70 mmHg, heart rate 84 times per minute, respiratory rate 24 times per minute, body temperature 36.5°C, and oxygen saturation 97% with room air. Physical examination revealed a lagging right chest movement and diminished tactile fremitus on the right and decreasing right vesicular breath sounds with no wheezing or rhonchi. Patient has no history of tuberculosis. We identified a palpable mass measuring 10 × 10 cm firm, fixed, without any skin decoloration.

The result of the initial chest X-ray was cavitating lesions with air-fluid level at right lower zone suggestive a lung abscess (with no significant changing compared to the previous X-ray) and minimal fibrosis in the left lower zone.

**Fig. 1:**
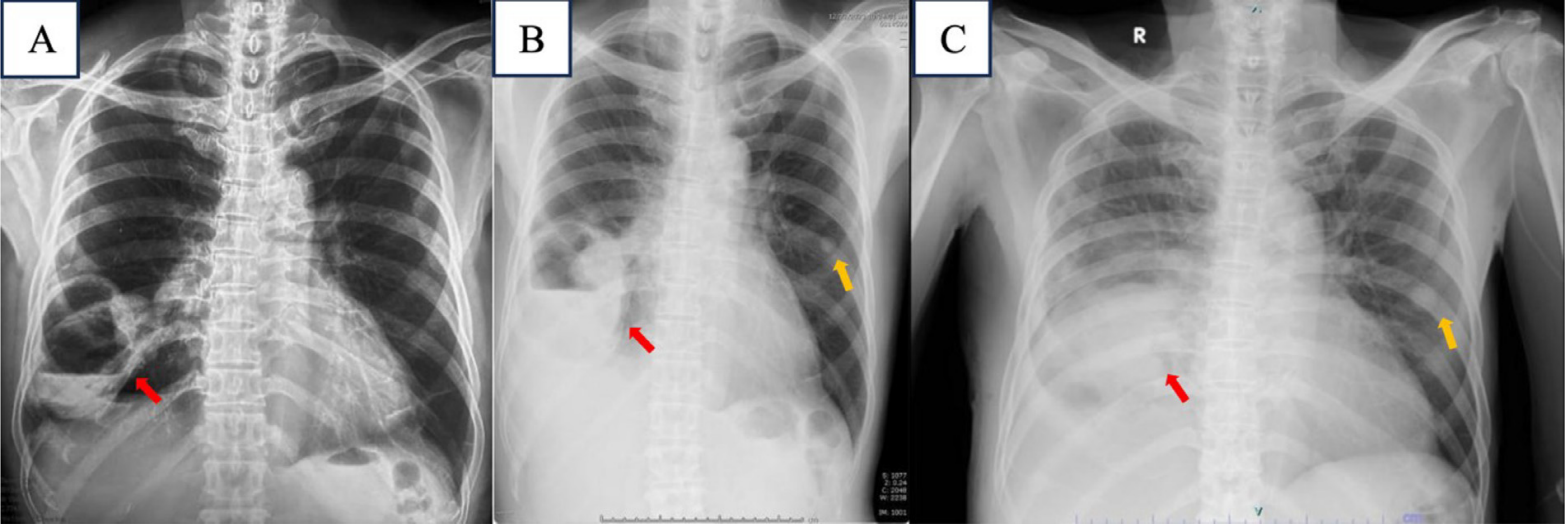
(A) Initial chest X-ray shows there is cavitating lesion with air-fluid level inside it (red arrow) (B) Second chest X-ray. The size of the lesion remains constant, but there is also right pleural effusion and nodule in left parahilar (yellow arrow) (C) and third chest X-ray before open biopsy shows, there is increase fluid level in the cavity and nodule in left parahilar (yellow arrow).

The patient has lost of follow-up because he felt much better after taking the medications, but then 4 months later he got the same illness and performed the second chest X-ray, chest MSCT and histopathology examination. From second chest X-ray, the cavitating lesion with air-fluid level was still remains with no significant size changing (Fig. 1).

**Fig. 2:**
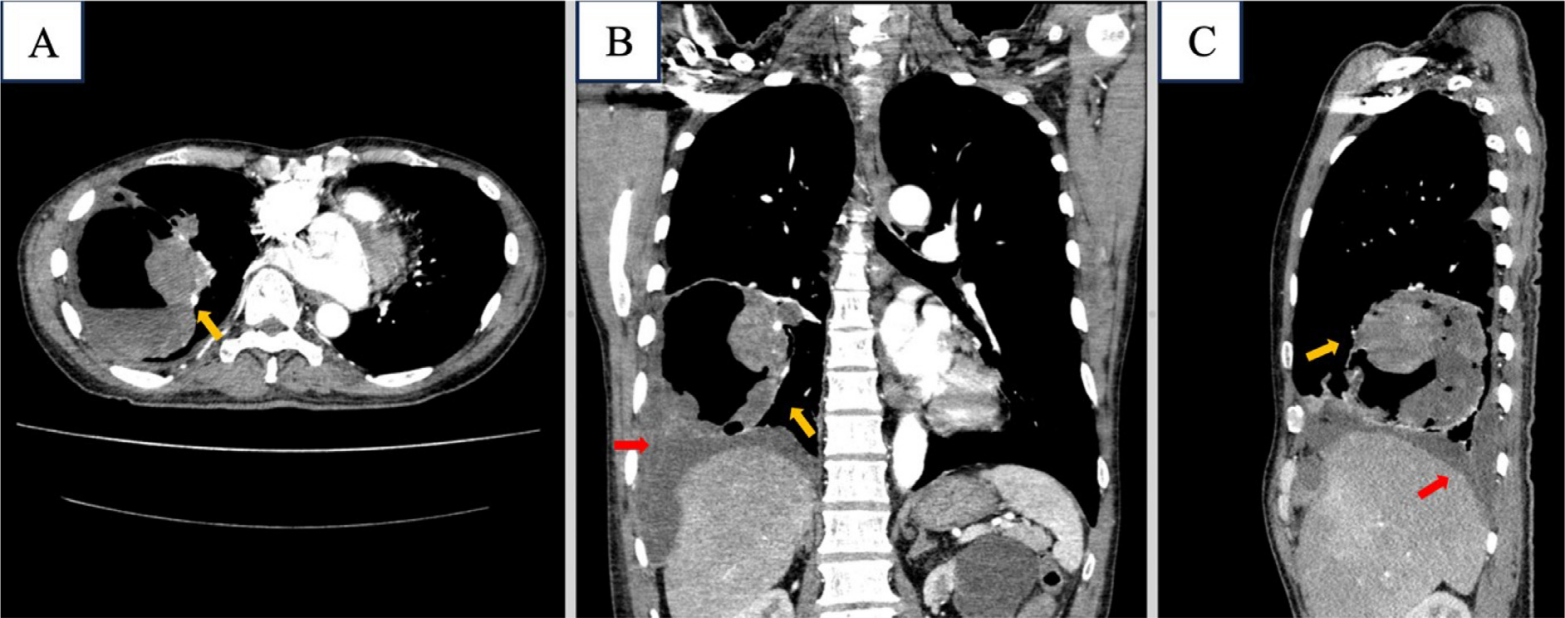
Contrast-enhanced CT-scan images of the chest region (A) Axial, (B) Coronal and (C) Sagital. Show cavitating lesion with solid and fluid components forming an air-fluid level (yellow arrow), in anterobasal segment inferior lobe of right lung and right pleural effusion (red arrow).

**Fig. 3:**
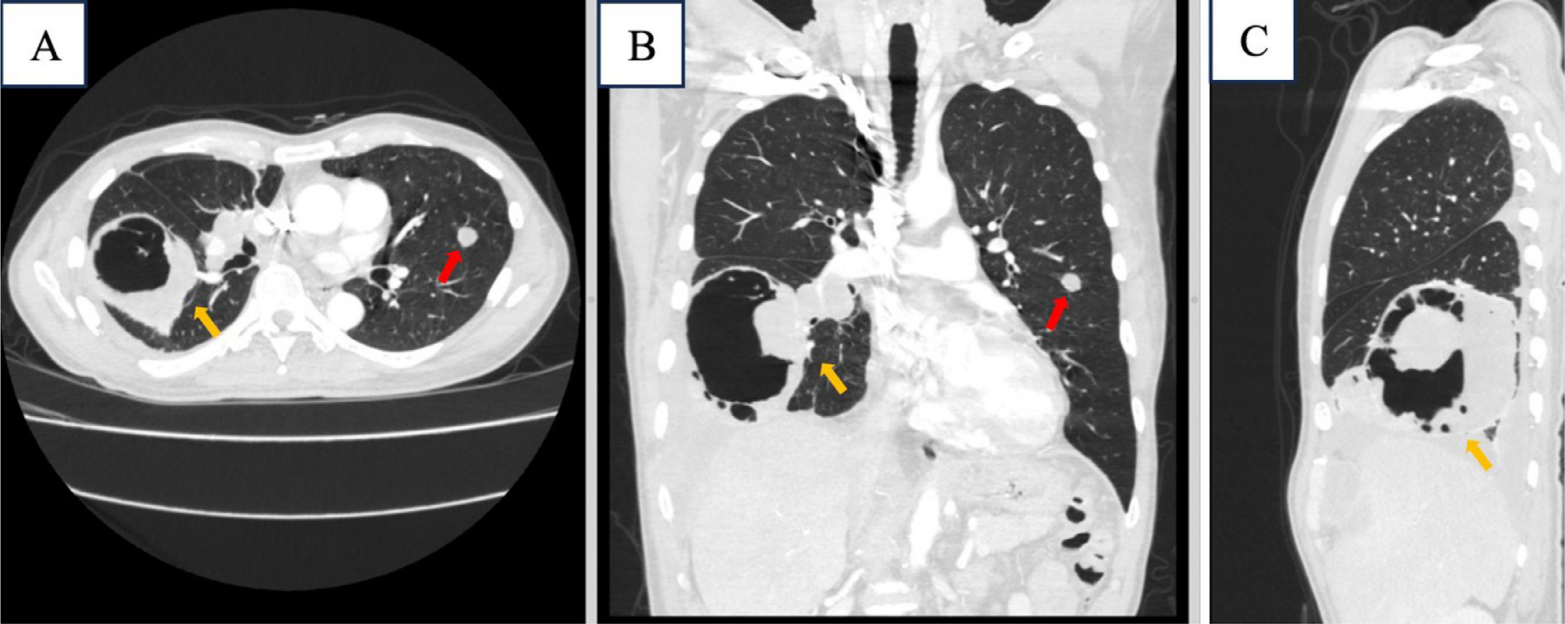
Contrast-enhanced CT-scan images of the chest region (A) Axial, (B) Coronal and (C) Sagittal. Show cavitating lesion with solid and fluid component forming an air-fluid level (yellow arrow) and nodule in superior lingula segment of the superior lobe of the left lung (red arrow).

**Fig. 4:**
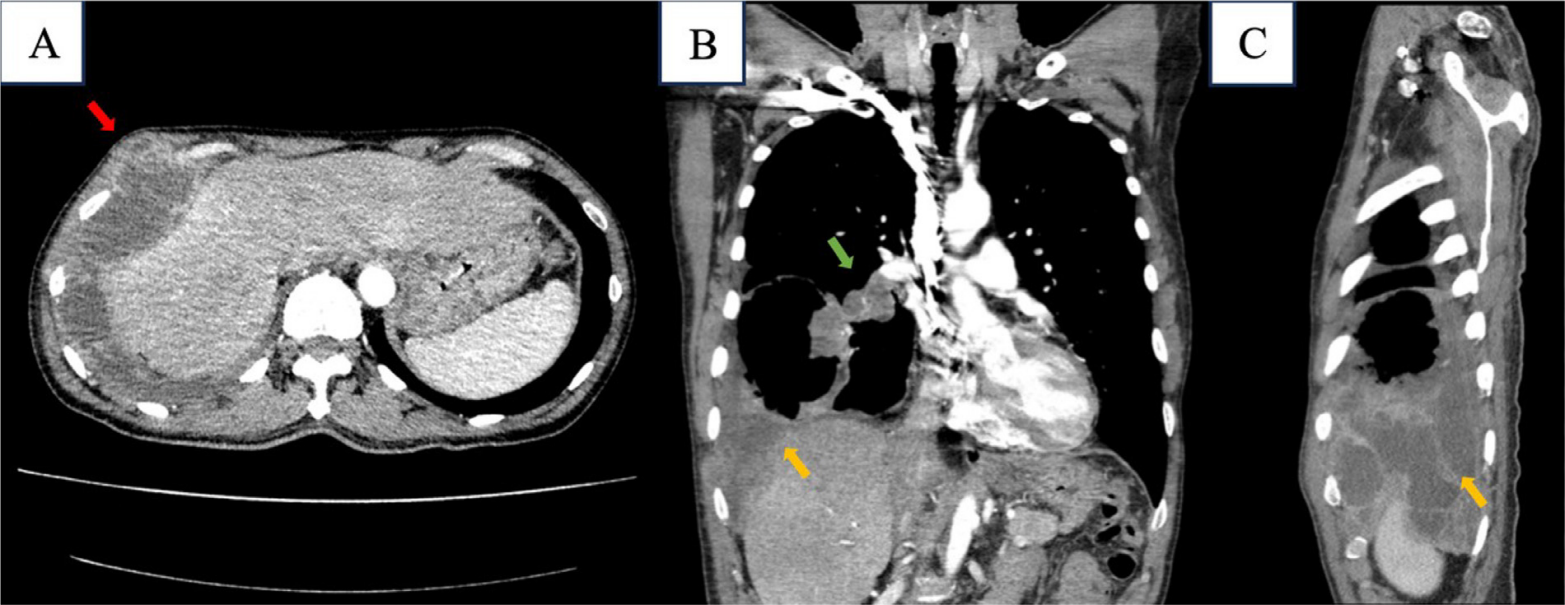
Contrast-enhanced CT-scan images of the chest region (A) Axial, (B) Coronal and (C) Sagittal. (A-C) Show loculated pleural effusion in right hemithorax (yellow arrow), some of which bulge to right anterior chest wall (red arrow), (C) Lymphnode enlargement at right peribronchial (green arrow).

Enhanced chest MSCT showed a cavitating lesion with solid-cystic component and air-fluid level, having 9.5 × 8.3 × 10.0 cm in size at anterobasal right inferior lung lobe an its solid part had a significant enhancement. We found a nodule measuring 1.2 × 1.2 cm at left superior lingula and loculated right pleural effusion. A right peribronchial lymphnode enlargement was identified with 3.4 × 3.1 cm in size (Fig. 2, Fig. 3, Fig. 4).

**Fig. 5:**
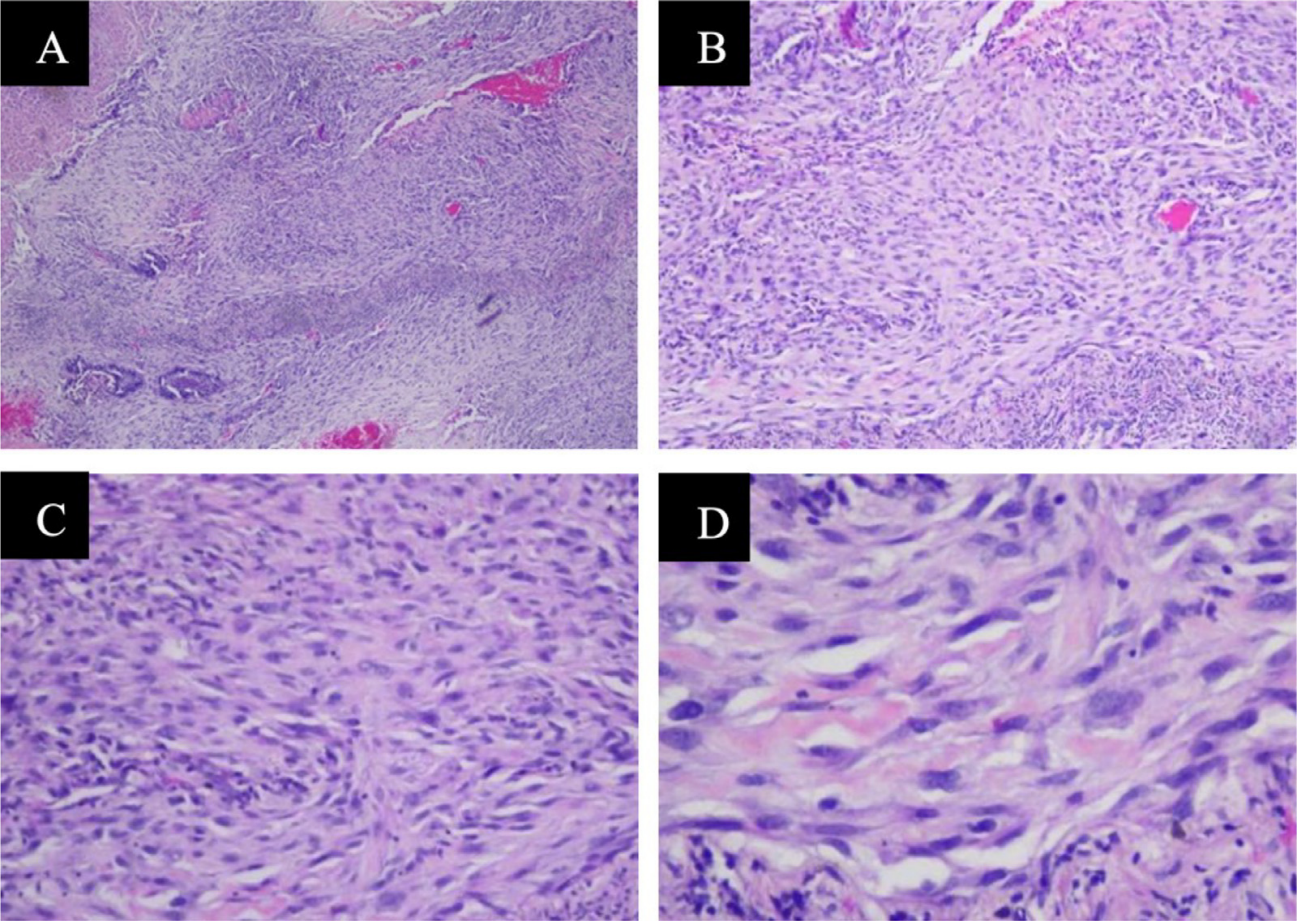
Open biopsy: Hematoxylin and eosin staining showed a rich variety of spindle cells (magnification, 40x, 100x, 200x and 400x) indicated a spindle cell mesenchymal tumor, suggesting a solitary fibrous tumor.

**Fig. 6:**
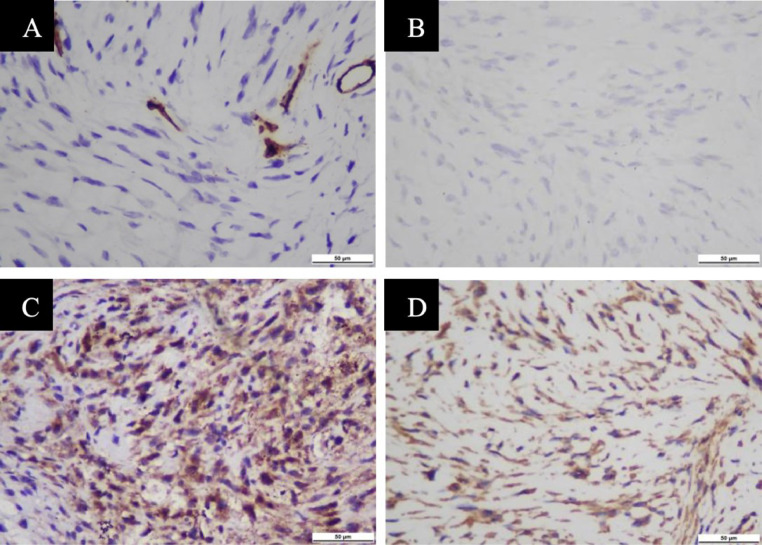
Open biopsy: Immunohistochemistry with vimentin, STAT-6,CD34, CD99 indicated a spindle cell mesenchymal tumor, suggesting a solitary fibrous tumor.

Cytological result from effusion was negative for malignancy, with ADA value of 14.4. Then followed by right thoracic wall mass fine needle biopsy, resulting in a metastatic carcinoma with differential diagnosis of mesothelioma. The multidiscipline team then decided to do an open biopsy due to its difficulty of having the representable sample from transthoracic core biopsy. The histological and IHC results were supported a spindle cell mesenchymal tumor. Other information we had from surgery, it was a massive adhesion between right inferior lung lobe and chest wall (Figs. 5 and 6).

## Discussion

SFT is a rare tumor that arises from mesenchymal cells, typically exhibits a hemangiopericytic pattern, and is classified as a soft tissue tumor with ambiguous histological characteristics and indeterminate malignancy. SFT has been categorized by the World Health Organization as a fibroblastic/myofibroblastic tumor. This tumor typically appears at the age of 20 to 70 years without any specific gender preference [3,4] and most frequently occurs in the pleura. The lung, central nervous system, kidney, and extrapleural SFT are rarely found. In the thoracic cavity, other than pleura, this tumor located in the mediastinum or intraparenchymal areas. Intrapulmonary SFT is atypical and usually asymptomatic [5]. Many theories have been proposed about intrapulmonary SFT: 1) Direct extension of subpleural mesenchyma with interlobular septa connective tissue, 2) Invagination of visceral pleura on its origin with mechanical pressure and causing intrapulmonary slow growth, and 3) Originally from pulmonary parenchymal fibroblasts with alveolar pneumocytes and bronchioles entrapment, not to be regarded as a sign of aggressiveness [6].

### Signs and symptoms

Depending on their size, solitary fibrous tumors of the thorax may cause no symptoms; most commonly, however, they may be associated with dyspnea or chest pain. The clinical presentation is characterized by a painless and gradually expanding mass, which is finally accompanied by symptoms of compression [1,2].

### Radiologic features

SFTs are described as well-defined tumors, oval or round in shape. Generally, a small solitary fibrous tumor of the thorax is well-defined and homogeneously hyperdense originating from the pleura. The hyperdensity of the mass is most likely due to their high collagen content with a rich capillary network. The larger tumors may have heterogeneous densities with various geographic enhancement patterns due to cystic degeneration, calcification, hemorrhage, and necrosis. Tumors with low attenuation are usually found in necrotic areas [1,7].

### Differential diagnosis

Intrapulmonary solitary fibrous tumors may present as well-defined round or ovoid nodules on CT, leading to a wide imaging differential diagnosis including hamartomas and carcinoid tumors [1]. Hamartoma is the third most common cause of a solitary pulmonary nodule, following granuloma and carcinoma. Calcification and fat components are specific findings in hamartomas. About 20% of carcinoids present as solitary pulmonary nodules in the lung periphery distal to the segmental bronchi, typically as slow-growing nodules. Atypical carcinoids are also usually peripheral, large, and well-circumscribed masses [6].

### Treatment

An adequate wedge resection, anatomic segmentectomy, and lobectomy, according to the location of the mass, are common surgical procedures for the resection of intra-pulmonary tumors. In the case of unresectable diseases, only radiotherapy or radio-chemotherapy may significantly ensure long-term local control of primary and metastatic lesions [9,10].

In this case, the initial diagnosis was abscess or lung cancer. The abscess was suspected due to the air-fluid level but this patient came with normal leukocyte levels, no foul-smelling breath, and the absence of other infection parameters so the abscess was ruled out. Lung cancer was considered due to the patient's age, tumor location, and its prevalence as a common lung cancer. The radiological features and symptoms were overlapped, requiring histopathological examination including immunohistochemistry for a definitive diagnosis due to the rarity of intraparenchymal SFT in the lungs.

The patient has final diagnosis of Solitary Fibrous Tumor (SFT) and it is exceptionally uncommon because of its air-fluid cavitation. Solitary fibrous tumors (SFT) typically appear as uniform solid masses on CT scans, although there have been cases of cystic masses, with well-defined borders and regular margins. The presence of a cavity, however, is an uncommon presentation in SFT. SFT is an intriguing tumor that poses challenges for physicians, surgeons, radiologists, and pathologists due to its often elusive diagnosis [3] because their appearance is similar to common lung tumors, so the diagnosis of intrapulmonary SFT presents unique challenges [8].

## Conclusion

Intrapulmonary solitary fibrous tumors (SFT), though rare, provide considerable diagnostic difficulties due to their diverse presentation and similarity to other pulmonary tumors. This case emphasizes the significance of a thorough diagnostic strategy, integrating clinical, radiologic, and pathologic assessments to diagnose SFT. Immunohistochemistry plays a crucial role in the diagnosis of this tumor. The intricacy and risk for misdiagnosis are shown by the presence of a cavitating lesion in this patient, which was initially mistaken for an abscess or cancer.

Due to the infrequency of intrapulmonary solitary fibrous tumors (SFT) and their tendency to resemble other lung tumors, early and accurate diagnosis is essential to guide appropriate management and avoid delays in definitive therapy. The primary treatment method remains surgical resection, with an approach according to the location and characteristics of the tumor.

This case contributes to the limited literature on intrapulmonary SFT, particularly those with cavitation. It emphasizes the importance of doctors being vigilant to accurately diagnose and treat this condition.

## Patient consent

Written informed consent was obtained from the patient for the publication of this case report.
